# Stainless-steel crowns in children: Norwegian and Finnish dentists’ knowledge, practice and challenges

**DOI:** 10.1186/s12903-021-01556-6

**Published:** 2021-04-12

**Authors:** M. M. Uhlen, B. Tseveenjav, E. Wuollet, J. Furuholm, V. Ansteinsson, A. Mulic, H. Valen

**Affiliations:** 1 Oral Health Centre of Expertise in Eastern Norway (OHCE-E), Oslo, Norway; 2 Department of Oral and Maxillofacial Diseases, University of Helsinki, Helsinki, Finland; 3 Nordic Institute of Dental Materials (NIOM), Oslo, Norway

**Keywords:** Stainless-steel crowns, Paediatric dentistry, Dental caries, Dental developmental disorders, Restorative dentistry, Treatment decisions

## Abstract

**Background:**

Stainless-steel crowns (SSCs) are recommended for restorative treatment of young teeth severely affected by caries, fractures or dental developmental disorders (DDDs). However, despite recommendations and clinical evidence, SSCs are not widely used by general dentists, who favour extraction and more conventional restorations. The present study aimed to investigate the views of and use of SSCs among Norwegian and Finnish dentists.

**Methods:**

The present study was a cross-sectional survey among Norwegian and Finnish dentists. An electronic questionnaire was sent to Norwegian and Finnish dentists asking whether they used SSCs and on which indications. In addition, the questionnaire assessed reasons for non-use and dentists’ perceptions regarding advantages and challenges in the use of SSCs, as well as the need for additional training. Distributions of background characteristics, use of and views on SSCs were calculated, and statistical significance of the associations between respondents’ background and their answers were evaluated.

**Results:**

Of the 574 Norwegian and 765 Finnish respondents, only 12.0% and 12.9% reported to use SSCs, respectively. The most frequently reported barrier reported by those who did not use SSCs was lack of practical training. The most frequent challenge reported by those using SSCs was difficulties in crown adjustment followed by aesthetic issues, and the most frequently reported advantage was that SSCs maintain the function and occlusion. The majority of respondents reported a need for more information and practical training in the use of SSCs, with hands-on course as their most frequently preferred education type.

**Conclusion:**

Although the value of SSCs for restoring young molars is recognized by Norwegian and Finnish dentists, SSCs are rarely used by general dentists. The majority of the respondents reported lack of training and materials and was interested in receiving more information and education.

## Background

The available treatment modalities for teeth severely affected by caries or dental developmental defects (DDDs) range from prevention to various forms of restorative treatment and extraction, and clinicians need to make the best treatment decision in both short- and long-term perspective [1]. Both patient- and tooth-related factors influence the decision, and treating severe lesions poses several technical challenges. Poor retention for restorations and possible contamination by saliva and blood require high clinical skills as well as patient cooperation [2]. Therefore, interventions may be challenging both for the dentist to perform and for children to cope with [3, 4].

Stainless-steel crowns (SSCs) are recommended for restorative treatment of severe multiple surface lesions of different origins, such as caries, fracture or DDDs [3]. Conventionally, complete caries removal and tooth preparation was considered necessary, usually requiring local anaesthesia. Later, the Hall Technique (HT) has been advocated as a less invasive biological approach using SSCs without caries removal or tooth preparation [5]. Due to the ongoing COVID-19 pandemic, which in all likelihood will continue to have a major impact on the practice of dentistry, avoidance of elective aerosol generating procedures has been recommended wherever possible. This aspect further increases the relevance of HT [6]. Although HT is supported by robust evidence and advocated for use by both general dentists and paediatric dentists, the technique has been met with some resistance [7].

In general, SSCs have consistently shown high success rates regardless of the technique used or type of study [8, 9]. Nevertheless, despite recommendations and clinical evidence, SSCs are not widely used by general dentists, who favour extraction and more conventional restorations, while paediatric dentists more frequently restore with SSCs [2, 10–13]. According to a survey among German dentists, the use of SSC is technically complex, cosmetically not acceptable, and the procedure time-consuming with low financial reimbursement [2].

A recent questionnaire study investigating treatment strategies and reasons for treatment choices among Norwegian and Finnish dentists, showed that SSCs were rarely chosen by general dentists when treating severe dental caries in primary teeth or DDDs in permanent teeth [14, 15]. Due to the high success rates of SSCs as well as their versatility, it would be of interest to gain more information about the reasons for the apparent reluctance of choosing SSCs. Therefore, the present study aimed to explore this finding further by investigating Norwegian and Finnish dentists’ current use and knowledge of SSCs as well as their challenges and need for further continuing education or training.

## Methods

The study was a part of a collaborative cross-sectional questionnaire study among dentists in Norway and Finland [14, 15]. A link to an electronic questionnaire was sent to dentists, including dental specialists, in the Public Dental Service (PDS) in Norway in May 2018 (n = 1,294) and to the members of the Finnish Dental Society in February 2019 (n = 3,840), using the software Questback Essential (Questback, Norway) [14, 15]. Replies were received from 614 Norwegian and 1,022 Finnish dentists, the response rates being 45.8% and 20.4%, respectively. After exclusion of those who did not do clinical work or treated children and respondents practicing in other countries, the total numbers of participants included were 574 and 765, respectively [14, 15]. The questionnaire was originally developed in Norwegian language, and later translated into Finnish and pilot-tested before distribution in Finland. Minor adjustments, such as counties, country of graduation and field of speciality, were made to suit local circumstances. Data on gender and age for all PDS-employed dentists in Norway were extracted from Statistics Norway, Dental Health [16]. The background information of the Finnish dentists was obtained from the Finnish Dental Association, which includes data of 95% of the dentists [17].

The questionnaire was anonymous and comprised three parts, of which the first was background information of the dentists. The second part was composed of three illustrated patient cases, and the respondents were asked to report the preferred treatment option in each case. The case descriptions and associated results have been reported previously [14, 15]. The third part of the questionnaire concentrated on the use of SSCs, and is reported in the present study.

The respondents were asked whether they used SSCs in clinical practice. Those who reported use of SSCs were asked on which indications they would use SSCs in primary dentition (“deep multi-surface caries”, “fractures”, “DDDs”, “other”), as well as in permanent dentition (“deep multi-surface caries”, “fractures”, “DDDs”, “endodontically treated molars”, “infractions”, “other”). Moreover, the same respondents were asked whether they had faced challenges related to the use of SSCs, by selecting from one or more of the following options: “Difficulties in crown adjustment”, “detachment of the crown”, “aesthetic issues”, “difficult procedure” and “allergies”. In addition, open commenting was possible, and the most frequent comments were summarised.

Those who reported not to use SSCs in their clinical practice were asked to report their perceived barriers against use of SSCs by selecting one or more of the following options: “Lack of knowledge about SSCs”, “lack of practical training on the use of SSCs”, “lack of materials/equipment”, or “other”.

All respondents were asked what they assume were the advantages of SSCs by selecting from one or more of the following options: “Maintains function and occlusion”, “keeps tooth symptomless”, “scientifically documented treatment”, “long durability” and “good prognosis”. The question was optional, and open commenting was possible. Concerning advantages of SSCs, the Norwegian dentists gave their answers in a prioritized order (Likert scale 1–5), while the Finnish dentists gave their answers by multiple choice.

Finally, the respondents were asked if they would like more information and practice regarded to the use of SSCs, and those who answered positively were asked to range the following educational forms by preference: “Lecture”, “illustrated guidelines”, “hands-on course”, “video demonstration”, “demonstration at your workplace” or “webinar”.

Data were processed and analysed using IBM SPSS version 25.0 (IBM Corp., Armonk, NY, USA). Frequency distributions were used for descriptive statistics. The statistical significance of the associations between respondents’ background and their answers were evaluated using Pearson Chi-Square test. The statistical significance was set at *p* < 0.05.

### Ethical consideration

The study participation was voluntary, without any compensation given to the respondents. Informed consent was obtained from all subjects. No personal information was gathered, and the anonymity was assured with Questback. The Norwegian Social Science Data Services approved the study design (Ref. No. 57710). All methods were performed in accordance with relevant guidelines and regulations in both countries.

## Results

Background characteristics of the dentists according to the use of SSCs are presented in Table 1.

**Table 1:** Norwegian and Finnish dentists’ background characteristics by use of stainless-steel crowns in their clinical practice

	Norway n = 574			Finland n = 765		
Background characteristics	Total N	SSC users n = 69 % (n)	Non-users n = 505 % (n)	Total N	SSC users n = 99 % (n)	Non-users n = 666 % (n)
Gender						
Female	447	11.0 (49)	89.0 (398)	618	12.9 (80)	87.1 (538)
Male	127	15.7 (20)	84.3 (107)	147	12.9 (19)	87.1 (128)
Age						
< 30	81	6.2 (5)	93.8 (76)	81	9.9 (8)	90.1 (73)
≥ 30	493	13.0 (64)	87.0 (429)	684	13.3 (91)	86.7 (593)
Country of graduation						
Nordic countries	447	12.3 (55)	87.7 (392)	724	13.0 (94)	87.0 (630)
Other countries	127	11.0 (14)	89.0 (113)	41	12.2 (5)	87.8 (36)
Year of graduation						
< 2000 (Finland)/ < 2001 (Norway)	189	17.5 (33)	82.5 (156)	454	10.8 (49)	89.2 (405)
≥ 2000 (Finland)/ ≥ 2001 (Norway)	385	9.4 (36)	90.6 (349)	311	16.1 (50)	83.9 (261)
Main working sector (> 50%)						
Public health care	568	12.0 (68)	88.0 (500)	578	14.2 (82)	85.8 (496)
Other (private, university)	6	16.7 (1)	83.3 (5)	187	9.1 (17)	90.9 (170)
Speciality						
No (general dentist)	553	11.0 (61)	89.0 (492)	710	10.6 (75)	89.4 (635)
Paediatric dentistry	7	100 (7)	–	10	100 (10)	–
Prosthetic dentistry	5	20.0 (1)	80.0 (4)	13	38.5 (5)	61.5 (8)
Orthodontics	4	–	100.0 (4)	10	20.0 (2)	80.0 (8)
Other	5	–	100.0 (5)	22	31.8 (7)	68.2 (15)

Of the respondents, 12.0% (n = 69) of the Norwegian and 12.9% (n = 99) of the Finnish dentists reported to use SSCs in their clinical practice. Use of SSCs was significantly associated with having a specialist degree in paediatric dentistry: While only approximately one tenth of the general dental practitioners in Norway and Finland reported use of SSCs, all of the specialists in paediatric dentistry in both countries used SSCs (p < 0.05).

### Clinical use of SSCs

“Severe multi-surface caries” was the most frequently chosen reason for SSC use in primary molars among Finnish dentists (90.9% (n = 90)) of those who reported to use SSCs. However, the same indication was chosen only by 46.4% (n = 32) of the Norwegian dentists who use SSCs (p < 0.05). Other indications were chosen followingly: «DDDs» by 75.4% (n = 52) of the Norwegian and 79.8% (n = 79) of the Finnish dentists, and «fractures» by 60.9% (n = 42) of the Norwegian and 66.7% (n = 66) of the Finnish dentists (p > 0.05). In the open comments, the most frequently reported reason for SSC use was “pulpotomized primary molars”, by one Norwegian and six Finnish dentists, respectively.

In permanent molars, “DDDs” was the most frequently chosen indication for SSCs, although it was significantly more common among Norwegian than Finnish dentists (85.5% (n = 59) and 70.7% (n = 70), respectively, (p < 0.05)). A significantly higher proportion of Norwegian dentists than Finnish dentists also chose “fractures” as reason for SSC use in permanent molars (56.5% (n = 39) and 33.3% (n = 33), respectively, (p < 0.05)), while no statistically significant difference was found in “severe multi-surface caries” between countries (34.8% (n = 24) of Norwegian and 35.4% (n = 35) of Finnish dentists). “Root-filled teeth” and “infractions” were reported by 11.6% (n = 8) and 8.7% (n = 6) of the Norwegian and 21.2% (n = 21) and 8.1% (n = 8) of the Finnish dentists, respectively. Temporary treatment of permanent molars was the most frequent reason mentioned among the open comments, reported by four Norwegian and two Finnish dentists, respectively.

### Perceived advantages of and challenges in the use of SSCs

**Fig. 1:**
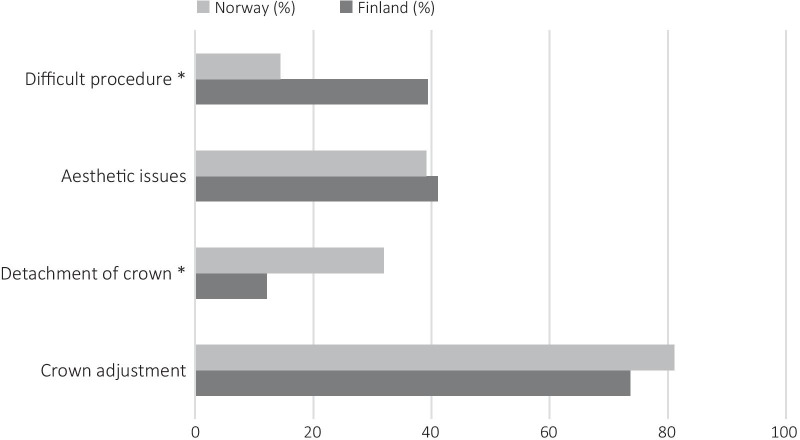
Self-perceived challenges in the use of stainless-steel crowns reported by Norwegian (n = 69) and Finnish (n = 99) dentists using SSCs in the clinic. *p < 0.05

The most frequently reported advantage of SSCs among both Norwegian (n = 307) and Finnish dentists (n = 708) was “SSCs maintain the function and occlusion” (77.5% (n = 238) and 97.3% (n = 689), respectively). Among the Finnish dentists, this was followed by “long durability”, chosen by 88.1% (n = 624) and “good prognosis”, chosen by 64.7% (n = 458). Among the Norwegian dentists, “SSCs keep the tooth symptomless” was chosen by 49.2% (n = 151), and “long durability”, chosen by 47.9% (n = 147). Among the open comments, the most frequently reported advantage of SSCs was that “it is an easy and time/resource saving treatment”, reported by 16 dentists. The most frequent challenge in the use of SSCs in clinical practice was reported to be “difficulties related to the adjustment of the crown” (81.1% (n = 56) of Norwegian and 73.7% (n = 73) of Finnish dentists using SSCs), followed by “aesthetic issues” (39.1% (n = 27) and 41.4% (n = 41), respectively) (Fig. 1). A significantly higher proportion of Norwegian (31.9%, n = 22) than Finnish dentists (12.1%, n = 12) found “detachment of the crown” to be a challenge, and a significantly higher proportion of Finnish (39.4%, n = 39) than Norwegian dentists (39.1%, n = 10) reported “difficult procedure” to be a challenge (*p* < 0.05). Only three Finnish dentists found “allergies” to be a challenge related to the use of SSCs. The most frequent challenge reported in the open comments was “patient cooperation”, reported by seven dentists.

### Personal and practical barriers against use of SSCs

**Fig. 2:**
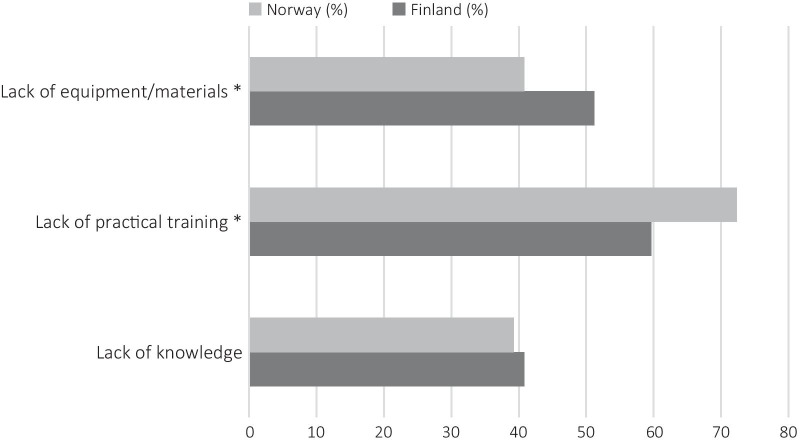
Barriers against use of stainless-steel crowns reported by Norwegian (n = 505) and Finnish (n = 666) dentists not using SSCs in the clinic. *p < 0.05

The most frequently reported barrier against use of SSCs among both Norwegian and Finnish dentists reporting not to use SSCs was “lack of practical training”; 72.3% (n = 365) and 59.2% (n = 394), respectively (Fig. 2) (*p* < 0.05). Of the Norwegian dentists, 40.8% (n = 206), and of the Finnish dentists, 51.2% (n = 341) reported “materials not available in the clinic” as a practical barrier against use (*p* < 0.05). “Lack of knowledge” was reported by 39.2% (n = 198) of the Norwegian and 40.8% (n = 272) of the Finnish dentists, respectively.

### Perceived need for more training/education

**Fig. 3:**
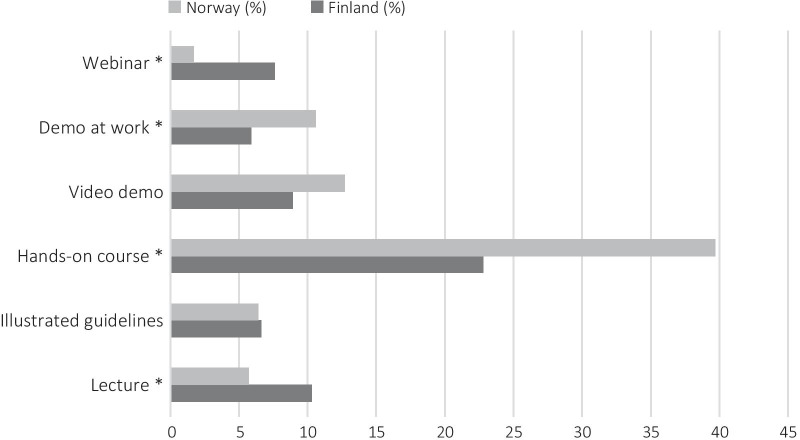
Type of training/education in the use of stainless-steel crowns preferred by Norwegian (n = 446) and Finnish dentists (n = 511) reporting that they would like more information and practical training in the use of SSCs. *p < 0.05

The majority of the respondents from both Norway (77.7%, n = 446) and Finland (66.8%, n = 511) reported that they would like more information and practical training in the use of SSCs. Figure 3 shows the preferred type of training/education. The most popular suggested type of education/training was hands-on course, chosen by 56.6% of the Norwegian and 43.4% of the Finnish dentists. A significantly higher proportion of Finnish than Norwegian dentists chose lecture and webinar as their preferred education form, while a significantly higher proportion of Norwegian than Finnish dentists would like hands-on course, video demonstration and clinical demonstration at their workplace (*p* < 0.05).

## Discussion

Despite recommendations and evidence of clinical benefits, SSCs seem to be an infrequent treatment choice among general dentists in the management of extensive caries in primary teeth and DDDs in permanent teeth [2, 14, 15]. According to dentists from both Norway and Finland, lack of practical training appears to be the most important barrier against SSC use. Although theoretical and practical education in the use of SSCs is implemented in the dental training in both countries, clinical practice during the education is limited, possibly due to low caries prevalence in the majority of the young population [18, 19]. This could contribute to the results from both countries showing that general dentists prefer other treatment options for severely carious or hypomineralised teeth, although Finnish dentists seem to adopt SSCs somewhat more than Norwegian dentists [14, 15]. Awareness of advantages seems to be related with the use of SSCs, according to the responses by Finnish dentists. In addition to the international recommendations, new national guidelines were recently published in Finland, recommending SSCs for extensively decayed primary teeth using both conventional technique and HT [20, 21]. There is a possibility that this has already impacted Finnish dentists’ attitudes toward SSCs. Although SSCs are a significant part of the specialist training in paediatric dentistry in Norway as well, SSCs are not specifically mentioned in the curricula for neither undergraduates nor paediatric specialist candidates in Norway. However, new national guidelines for dental restorations in children are in progress, and SSCs are mentioned in the draft document [22].

In both Norway and Finland, health care is predominantly publicly funded or subsidized, and dental health care services are provided free of charge for children under 18 years old in the public dental service (PDS). Therefore, while treatment costs have been reported as a main reason for the infrequent use of SSCs in countries where patients pay for the treatment themselves, this is less relevant in Norway and Finland [2, 10]. Still, the costs of SSCs were also raised in open comments of this survey and may partly explain why lack of materials was reported as the second most frequent reason for not using SSCs.

According to the present study, paediatric dentists in Norway and Finland seem to use SSCs more frequently than general dentists, which is in line with results from recent studies in other countries [12, 13, 23–25]. This difference is most likely due to the reported lack of training among general dentists. During postgraduate education in paediatric dentistry, SSCs constitute a larger proportion of the curriculum and a higher number of suitable patient cases than in the undergraduate education, due to referrals of patients with severe caries and DDDs as well as children with special needs, complex dental problems or medical conditions which may impact oral health. The specializing dentists are thus provided with more hands-on training under supervision than undergraduates. Moreover, they more frequently treat children under general anaesthesia. Indeed, SSCs are more frequently used in general anaesthesia service [11].

Corresponding to results from other studies, general dentists in Norway and Finland seem to prefer more conventional and less interventionist treatment options above SSCs for the treatment of severe caries in primary molars and of permanent molars with DDDs [14, 15]. Norwegian dentists have previously been reluctant to remove tooth substance in general [26]. Kopperud et al. have suggested that it is likely that many dentists have used the approach of minimal invasive dentistry too strict in cases where a more invasive approach may be needed, such as in treatment of molar-incisor hypomineralization (MIH) [27]. Another explanation for limited tooth substance removal in severe MIH cases could be that dentists find the patient group challenging to treat because of insufficient pain control and associated behavioural management problems [27]. However, Norwegian and Finnish dentists have been shown to highly individualize their treatment strategies for each patient when treating children and adolescents [14, 15].

In the UK, the use of SSCs has increased after HT has gained popularity [28, 29]. SSCs placed using HT have shown a better success rate than conventional restorations in decayed primary teeth [3, 5, 30, 31]. Moreover, there is a lower risk of pain when using SSCs with HT compared to other fillings [21]. The HT may not be a commonly known treatment in Norway and Finland. However, it has recently been introduced in the curriculum in the undergraduate program in Finland [20]. In the future, it may be evaluated whether the use of SSCs in Norway and Finland increases if HT becomes a more commonly known procedure. Seen from the above-mentioned perspective, this might be a technique dentists could be expected to embrace if given the opportunity to learn and be confident using it. In addition, in light of the ongoing COVID-19 outbreak, use of HT is more relevant than ever [6].

The majority of both Norwegian and Finnish dentists in this survey reported the adjustment of the crown to be challenging. Aesthetic requirements were the second most frequently reported challenge in the use of SSCs, although the same study sample reported aesthetics to be among the least important factors affecting treatment choice in children [14, 15]. Poor aesthetics have been reported as limitations of SSCs by dental practitioners in other studies [2, 10]. However, a study investigating the child and parental views on SSCs found that SSCs were viewed favourably by most children and their parents, and that the majority expressed little or no concern about their appearances. The authors pointed out that these findings may encourage clinicians who have been reluctant to use SSCs due to aesthetic concerns [32].

Given that lack of practical training was the most frequently reported reason for non-use of SSCs in this study, it is not surprising that the majority of respondents reported a need for more information and education in the use of SSCs. Along with the identified perception of advantages of SSCs, this indicates motivation and interest among clinicians. The majority of the respondents chose hands-on course as their preferred education format, and based on the generally low caries prevalence among children in Norway and Finland today, this is a comprehensive finding which supports the lack of suitable clinical training.

The gender and age distribution of the Norwegian respondents reflected the age distribution of dentists working in the PDS. The study sample from Finland also resembled the background population in most aspects, but dentists younger than 30 years were over-represented and dentists older than 60 years were under-represented compared to the background population. As expected, the number of dentists having a postgraduate speciality was low, and this should be considered when interpreting the results. In addition, there were more respondents from North and East Finland than what the distribution of the background population would assume.

It may be argued that the reported attitudes and routines do not necessarily reflect actual behaviour. The present study was questionnaire-based, and study participants self-selected to complete the survey, thus answer bias related to personal interests of clinicians may have occurred. In addition, in this kind of survey, it is possible that respondents adapt their answers to public opinion instead of responding realistically. This should therefore be considered in interpreting the findings.

Apparently, dentists do not necessarily act in accordance with national recommendations or even their own perception of the benefit of SSCs when treating children with extensively decayed primary teeth. This indicates a gap between self-perceived knowledge and practice, and recommendations of paediatric clinical best practice guidelines on the use of SSCs.

In clinical cases where SSCs are recommended, general dentists in Norway and Finland mostly prefer other, more conventional treatment options [15]. However, one should bear in mind that if dentists and patients overcome the technical difficulties and the aesthetic issues, in a long-term perspective, SSCs have a high success rate and a longer durability compared to conventional direct restorations, which often require retreatment and renewal [11]. SSCs are thereby cost-effective in the long-term, although the initial costs and efforts needed seem to be high. More frequent use may be achieved by enhanced education and information, which eventually may be advantageous for both patients’ experience with dental settings as well as for economic considerations.

## Conclusions

Although Norwegian and Finnish dentists apparently understand the value of SSCs for restoring young molars and improving patients’ oral health, SSCs are an infrequent treatment choice among general dentists. The majority of respondents reported lack of training and materials and was interested in receiving more information and education. Therefore, more focus on SSCs in the curricula and more information and practical training of general dentists should be established. This may result in a higher confidence among dentists to use SSCs that ultimately may lead to an increased use of SSCs when indicated.

## Data Availability

The datasets used and/or analysed during the current study available from the corresponding author on reasonable request.

## Abbreviations

DDD: Dental developmental disorder
HT: Hall technique
MIH: Molar-incisor hypomineralization
PDS: Public dental service
SSC: Stainless-steel crown

## References

[1] Elhennawy K, Schwendicke F (2016). Review article: Managing molar-incisor hypomineralization: a systematic review. J Dent.

[2] Santamaría RM, Pawlowitz L, Schmoeckel J, Alkilzy M, Splieth CH (2018). Use of stainless steel crowns to restore primary molars in Germany: questionnaire-based cross-sectional analysis. Int J Paediatr Dent.

[3] Innes NP, Ricketts D, Chong LY, Keightley AJ, Lamont T, Santamaria RM. Preformed crowns for decayed primary molar teeth. Cochrane Database Syst Rev. 2015(12):Cd005512. 10.1002/14651858.CD005512.pub3 PMC7387869 26718872

[4] Innes NP, Evans DJ (2013). Modern approaches to caries management of the primary dentition. Br Dent J.

[5] Innes NP, Evans DJ, Stirrups DR (2007). The Hall Technique; a randomized controlled clinical trial of a novel method of managing carious primary molars in general dental practice: acceptability of the technique and outcomes at 23 months. BMC Oral Health.

[6] Al-Halabi M, Salami A, Alnuaimi E, Kowash M, Hussein I (2020). Assessment of paediatric dental guidelines and caries management alternatives in the post COVID-19 period. A critical review and clinical recommendations. Eur Arch Paediatr Dent.

[7] Hussein I, Al Halabi M, Kowash M, Salami A, Ouatik N, Yang YM, Duggal M, Chandwani N, Nazzal H, Albadri S, Roberts A, Al-Jundi S, Nzomiwu C, El Shahawy O, Attaie A, Mohammed O, Al-Sane M (2020). Use of the Hall technique by specialist paediatric dentists: a global perspective. Br Dent J.

[8] Randall RC, Vrijhoef MM, Wilson NH (2000). Efficacy of preformed metal crowns vs. amalgam restorations in primary molars: a systematic review. J Am Dent Assoc.

[9] Chompu-inwai P, Boonsongsawat K, Sastraruji T, Sophasri T, Mankaen S, Nondon S (2015). Three incomplete caries removal techniques compared over two years in primary molars with asymptomatic deep caries or reversible pulpitis. Pediatr Dent.

[10] Threlfall AG, Pilkington L, Milsom KM, Blinkhorn AS, Tickle M (2005). General dental practitioners' views on the use of stainless steel crowns to restore primary molars. Br Dent J.

[11] Tseveenjav B, Furuholm J, Mulic A, Valen H, Maisala T, Turunen S (2018). Survival of extensive restorations in primary molars: 15-year practice-based study. Int J Paediatr Dent.

[12] Barker AM, Mathu-Muju KR, Nash DA, Li H-F, Bush HM (2012). Practice patterns of general dentists treating children in Kentucky: implications for access to care. Pediatr Dent.

[13] Lee GHM, McGrath C, Yiu CKY (2013). The care of the primary dentition by general dental practitioners and paediatric dentists. Int Dent J.

[14] Uhlen MM, Valen H, Karlsen LS, Skaare AB, Bletsa A, Ansteinsson V (2019). Treatment decisions regarding caries and dental developmental defects in children—a questionnaire-based study among Norwegian dentists. BMC Oral Health.

[15] Wuollet E, Tseveenjav B, Furuholm J, Waltimo-Sirén J, Valen H, Mulic A (2020). Restorative material choices for extensive carious lesions and hypomineralisation defects in children: a questionnaire survey among Finnish dentists. Eur J Paedriat Dent.

[16] Statistisk sentralbyrå (SSB). 11774: Årsverk i offentlig og privat tannhelsetjeneste, etter region, personellgruppe, sektor, statistikkvariabel og år. https://www.ssb.no/statbank/table/11774/tableViewLayout1/. Accessed 20 May 2020.

[17] Helsinki: The Finnish Dental Association, 2018. Statistics of dentists by hospital districts. https://www.hammaslaakariliitto.fi/fi/liiton-toiminta/tutkimukset-ja-tilastot/tilastot/hammaslaakarit-sairaanhoitopiireittain#.XMbHcgzaUk. Accessed 15 Oct 2020.

[18] Vehkalahti M, Tarkkonen L, Varsio S, Heikkilä P (1997). Decrease in and polarization of dental caries occurrence among child and youth populations, 1976–1993. Caries Res.

[19] Wigen TI, Wang NJ (2010). Caries and background factors in Norwegian and immigrant 5-year-old children. Community Dent Oral Epidemiol.

[20] Working group set up by the Finnish Medical Society Duodecim and the Finnish Dental Society Apollonia. Tooth restoration: Current Care Guidelines. www.kaypahoito.fi. Accessed Oct 15, 2020.

[21] Innes NPT. Preformed crowns for decayed primary molar teeth. Cochrane Database Syst Rev. 2015(12). 10.1002/14651858.CD005512.pub3 PMC7387869 26718872

[22] The Norwegian Directorate of Health. Tannhelsetjenester til barn og unge 0–20 år—Del 2 (høringsutkast). https://www.helsedirektoratet.no/retningslinjer/tannhelsetjenester-til-barn-og-unge-0-20-ar-del-2-horingsutkast/operativ-behandling-av-karies-hos-barn-og-unge. Accessed 15 Oct 2020.

[23] Dastouri M, Kowash M, Al-Halabi M, Salami A, Khamis AH, Hussein I (2020). United Arab Emirates dentists' perceptions about the management of broken down first permanent molars and their enforced extraction in children: a questionnaire survey. Eur Arch Paediatr Dent.

[24] Taylor GD, Pearce KF, Vernazza CR (2019). Management of compromised first permanent molars in children: cross-sectional analysis of attitudes of UK general dental practitioners and specialists in paediatric dentistry. Int J Paediatr Dent.

[25] Crombie FA, Manton DJ, Weerheijm KL, Kilpatrick NM (2008). Molar incisor hypomineralization: a survey of members of the Australian and New Zealand Society of Paediatric Dentistry. Aust Dent J.

[26] Vidnes-Kopperud S, Tveit AB, Espelid I (2011). Changes in the treatment concept for approximal caries from 1983 to 2009 in Norway. Caries Res.

[27] Kopperud SE, Pedersen CG, Espelid I (2016). Treatment decisions on molar-incisor hypomineralization (MIH) by Norwegian dentists—a questionnaire study. BMC Oral Health.

[28] Seale NS, Randall R (2015). The use of stainless steel crowns: a systematic literature review. Pediatr Dent.

[29] Roberts A, McKay A, Albadri S (2018). The use of Hall technique preformed metal crowns by specialist paediatric dentists in the UK. Br Dent J.

[30] Chisini LA, Collares K, Cademartori MG, de Oliveira LJC, Conde MCM, Demarco FF (2018). Restorations in primary teeth: a systematic review on survival and reasons for failures. Int J Paedr Dent.

[31] Santamaria RM, Innes NPT, Machiulskiene V, Evans DJP, Splieth CH (2014). Caries management strategies for primary molars: 1-yr randomized control trial results. J Dent Res.

[32] Bell SJ, Morgan AG, Marshman Z, Rodd HD (2010). Child and parental acceptance of preformed metal crowns. Eur Arch Paedriatr Dent.

[33] Lovdata. Lov om medisinsk og helsefaglig forskning (helseforskningsloven). Kapittel 3. Søknad og meldeplikt til den regionale komiteen for medisinsk og helsefaglig forskningsetikk (§§9–12). https://lovdata.no/dokument/NL/lov/2008-06-20-44#KAPITTEL_3. Accessed 16 Mar 2021.

[34] Regional Commitee for Medical and Health Research Ethics. Rules and procedures. https://helseforskning.etikkom.no/reglerogrutiner/soknadsplikt/sokerikkerek?p_dim=34999&_ikbLanguageCode=us. Accessed 16 Mar 2021.

[35] Norwegian Centre for Research Data. https://www.nsd.no/en/about-nsd-norwegian-centre-for-research-data/. Accessed 16 Mar 2021.

